# Supplementary material for: The First Plastid Genome of the Holoparasitic Genus Prosopanche (Hydnoraceae)
Source: Plants (Basel). 2020 Mar 1;9(3):306. doi: 10.3390/plants9030306 (PMC7154897; doi:10.3390/plants9030306)
Supplement: Supplementary file 1 [file plants-09-00306-s001.zip › supporting material final proofreading/Table S1.pdf]

tRNA	Anticodon predicted isotype	Predicted Anticodon	Anticodon v. Isotype Model	Ala	Arg	Asn	Asp	Cys	Gln	Glu	Gly	His	Ile	Leu	Lys	Met	Phe	Pro	SeC	Ser	Thr	Trp	Tyr	Val	iMet
trnE-UUC	Glu	TTC	Inconsistent	9.4	no hit	no hit	14.0	no hit	16.4	39.8	29.6	60.1	no hit	no hit	no hit	no hit	no hit	-0.5	no hit	no hit	31.4	no hit	no hit	20.0	no hit
trnfM-CAU	Met	CAT	Consistent	38.5	37.8	38.5	-2.5	39.3	17.7	23.2	28.7	37.7	45.5	9.2	43.0	63.9	34.8	27.1	no hit	0.7	40.9	33.9	38.8	40.9	14.0
trnI-CAU	Met	CAT	Inconsistent	25.6	40.5	17.4	7.4	30.5	27.1	-10.7	29.7	35.8	28.0	20.3	2.1	14.5	-3.2	-16.5	no hit	17.4	55.3	27.2	34.5	17.8	no hit
trnW-CCA	Cys	ACA	Inconsistent	22.5	12.4	-0.1	no hit	14.4	6.0	no hit	3.6	18.9	28.6	no hit	5.6	12.5	2.5	5.2	no hit	no hit	32.4	24.5	13.7	20.4	no hit
trnY-GUA	Tyr	GTA	Consistent	18.9	no hit	-6.8	no hit	51.5	37.7	no hit	14.6	29.9	30.4	39.6	-5.9	no hit	-13.5	-12.5	-16.4	54.9	39.8	10.4	63.3	4.6	no hit

Top score

2nd highest score

3rd highest score
