# Supplementary material for: The First Plastid Genome of the Holoparasitic Genus Prosopanche (Hydnoraceae)
Source: Plants (Basel). 2020 Mar 1;9(3):306. doi: 10.3390/plants9030306 (PMC7154897; doi:10.3390/plants9030306)
Supplement: Supplementary file 1 [file plants-09-00306-s001.zip › supporting material final proofreading/Table S2.pdf]

| Species | Gene | G3s | C3s | A3s | T3s | GC |
|---|---|---|---|---|---|---|
| *P. americana* | accD | 0.0385 | 0.0711 | 0.6904 | 0.5803 | 0.234 |
| *H. visseri* | | 0.0391 | 0.0545 | 0.5934 | 0.6683 | 0.237 |
| *A. contorta* | | 0.274 | 0.1885 | 0.3449 | 0.5585 | 0.366 |
| *P. americana* | rpl14 | 0.0395 | 0.0309 | 0.5361 | 0.6392 | 0.240 |
| *H. visseri* | | 0.0128 | 0.0404 | 0.6495 | 0.5455 | 0.242 |
| *A. contorta* | | 0.254 | 0.1881 | 0.4896 | 0.4059 | 0.404 |
| *P. americana* | rpl16 | 0.0211 | 0.0396 | 0.6091 | 0.5644 | 0.299 |
| *H. visseri* | | 0.0333 | 0.0505 | 0.6667 | 0.4848 | 0.282 |
| *A. contorta* | | 0.262 | 0.2130 | 0.4865 | 0.3611 | 0.453 |
| *P. americana* | rpl2 | 0.0417 | 0.0305 | 0.6510 | 0.5228 | 0.286 |
| *H. visseri* | | 0.0537 | 0.0704 | 0.6575 | 0.4975 | 0.280 |
| *A. contorta* | | 0.317 | 0.1983 | 0.4118 | 0.3966 | 0.444 |
| *P. americana* | rpl36 | 0.0435 | 0.0000 | 0.6552 | 0.6154 | 0.207 |
| *H. visseri* | | 0.1667 | 0.1250 | 0.6333 | 0.4167 | 0.243 |
| *A. contorta* | | 0.306 | 0.1034 | 0.4194 | 0.4138 | 0.405 |
| *P. americana* | rps11 | 0.0233 | 0.0189 | 0.5833 | 0.6132 | 0.235 |
| *H. visseri* | | 0.0288 | 0.0420 | 0.6803 | 0.4958 | 0.239 |
| *A. contorta* | | 0.241 | 0.1417 | 0.4615 | 0.3917 | 0.464 |
| *P. americana* | rps12 | 0.0426 | 0.0505 | 0.6075 | 0.5152 | 0.292 |
| *H. visseri* | | 0.1250 | 0.0213 | 0.5670 | 0.5319 | 0.319 |
| *A. contorta* | | 0.270 | 0.1942 | 0.4519 | 0.4078 | 0.428 |
| *P. americana* | rps14 | 0.0597 | 0.0133 | 0.7561 | 0.4533 | 0.204 |
| *H. visseri* | | 0.0548 | 0.0541 | 0.6835 | 0.5135 | 0.212 |
| *A. contorta* | | 0.381 | 0.1772 | 0.4024 | 0.3418 | 0.447 |
| *P. americana* | rps18 | 0.0690 | 0.0303 | 0.7671 | 0.4394 | 0.134 |
| *H. visseri* | | 0.0926 | 0.0794 | 0.6418 | 0.6111 | 0.170 |
| *A. contorta* | | 0.288 | 0.1786 | 0.4091 | 0.4524 | 0.380 |
| *P. americana* | rps19 | ***** | ***** | ***** | ***** | ***** |
| *H. visseri* | | 0.0000 | 0.0678 | 0.7419 | 0.5085 | 0.214 |
| *A. contorta* | | 0.307 | 0.1892 | 0.4286 | 0.4189 | 0.384 |
| *P. americana* | rps2 | 0.0272 | 0.0189 | 0.7005 | 0.5991 | 0.144 |
| *H. visseri* | | 0.0828 | 0.0789 | 0.6436 | 0.5877 | 0.194 |
| *A. contorta* | | 0.287 | 0.1546 | 0.4114 | 0.4485 | 0.397 |
| *P. americana* | rps3 | 0.0439 | 0.0449 | 0.6733 | 0.6026 | 0.163 |
| *H. visseri* | | 0.0556 | 0.0563 | 0.6582 | 0.5500 | 0.206 |
| *A. contorta* | | 0.272 | 0.1677 | 0.4798 | 0.4311 | 0.376 |
| *P. americana* | rps4 | 0.0374 | 0.0635 | 0.7323 | 0.5476 | 0.168 |
| *H. visseri* | | 0.0577 | 0.0484 | 0.6560 | 0.6048 | 0.207 |
| *A. contorta* | | 0.254 | 0.1595 | 0.4540 | 0.4479 | 0.393 |
| *P. americana* | rps7 | 0.0244 | 0.0317 | 0.7552 | 0.5238 | 0.179 |
| *H. visseri* | | ***** | ***** | ***** | ***** | ***** |
| *A. contorta* | | 0.207 | 0.1417 | 0.5231 | 0.4250 | 0.406 |
| *P. americana* | rps8 | 0.0462 | 0.0532 | 0.6404 | 0.5638 | 0.185 |
| *H. visseri* | | 0.0563 | 0.0323 | 0.6522 | 0.5699 | 0.204 |
| *A. contorta* | | 0.260 | 0.1161 | 0.4369 | 0.4375 | 0.369 |
| *P. americana* | ycf1 | 0.0499 | 0.0573 | 0.6924 | 0.6081 | 0.174 |
| *H. visseri* | | 0.0573 | 0.0479 | 0.6761 | 0.6133 | 0.194 |
| *A. contorta* | | 0.245 | 0.1646 | 0.5186 | 0.4803 | 0.319 |
| *P. americana* | ycf2 | 0.0477 | 0.0463 | 0.6994 | 0.6345 | 0.131 |

| | | | | | | | |
|---|---|---|---|---|---|---|---|
| *H. visseri* | | | 0.0698 | 0.0625 | 0.6663 | 0.6019 | 0.170 |
| *A. contorta* | | | 0.346 | 0.2093 | 0.3949 | 0.4403 | 0.381 |
