# Supplementary figures and images for: The First Plastid Genome of the Holoparasitic Genus Prosopanche (Hydnoraceae)
Source: Plants (Basel). 2020 Mar 1;9(3):306. doi: 10.3390/plants9030306 (PMC7154897; doi:10.3390/plants9030306)

trnE-UUC

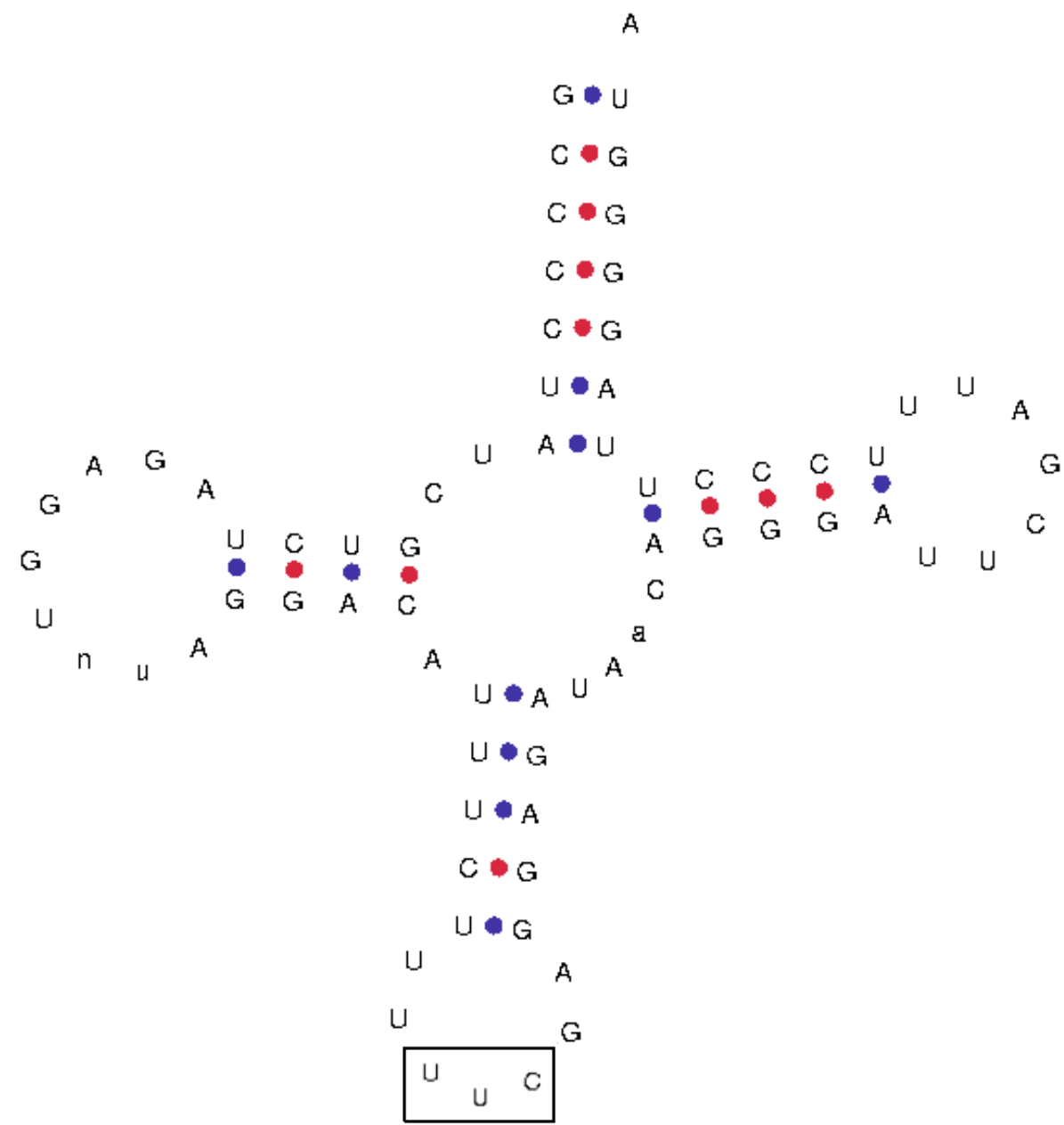

trnfM-CAU

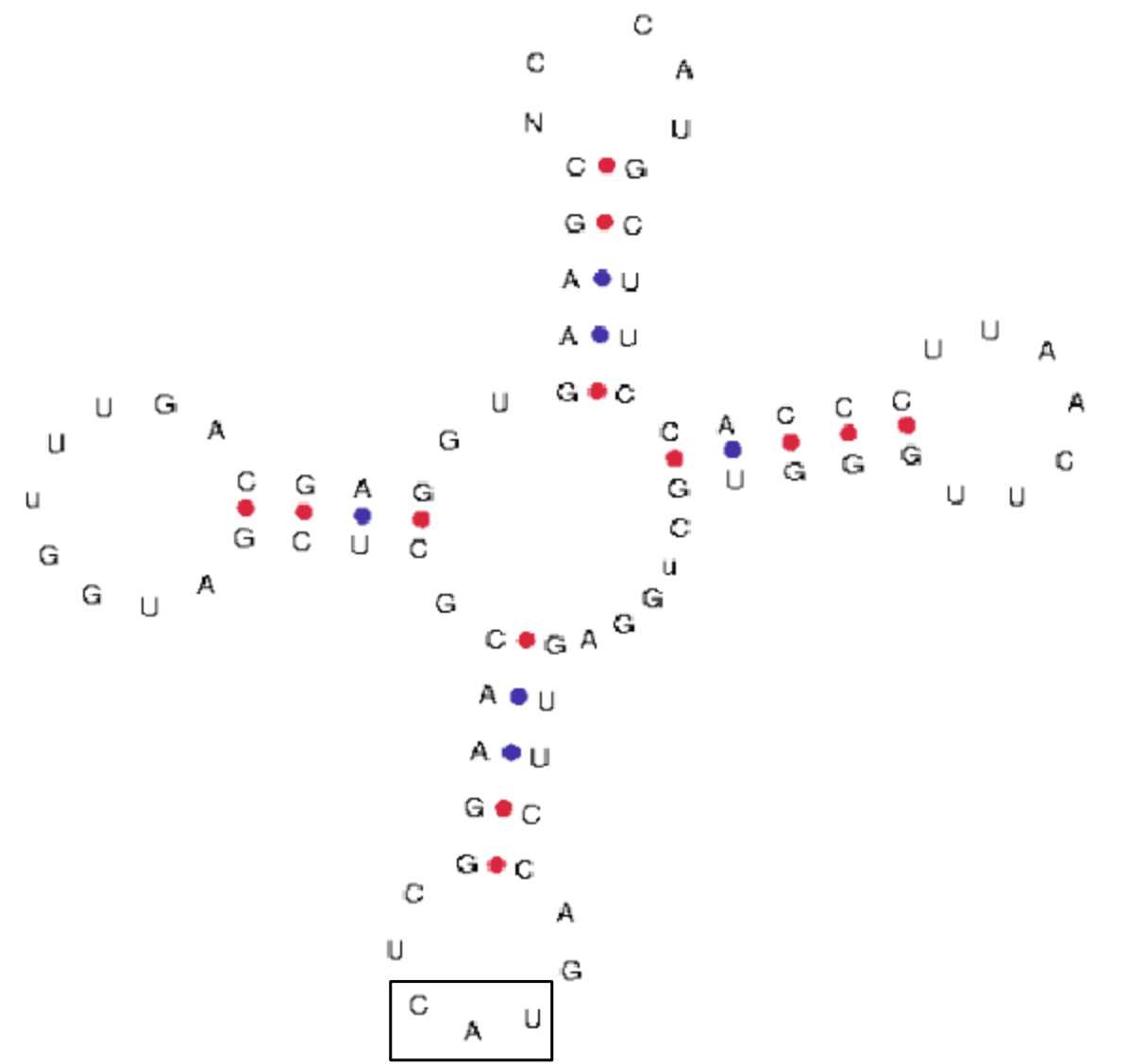

trnI-CAU

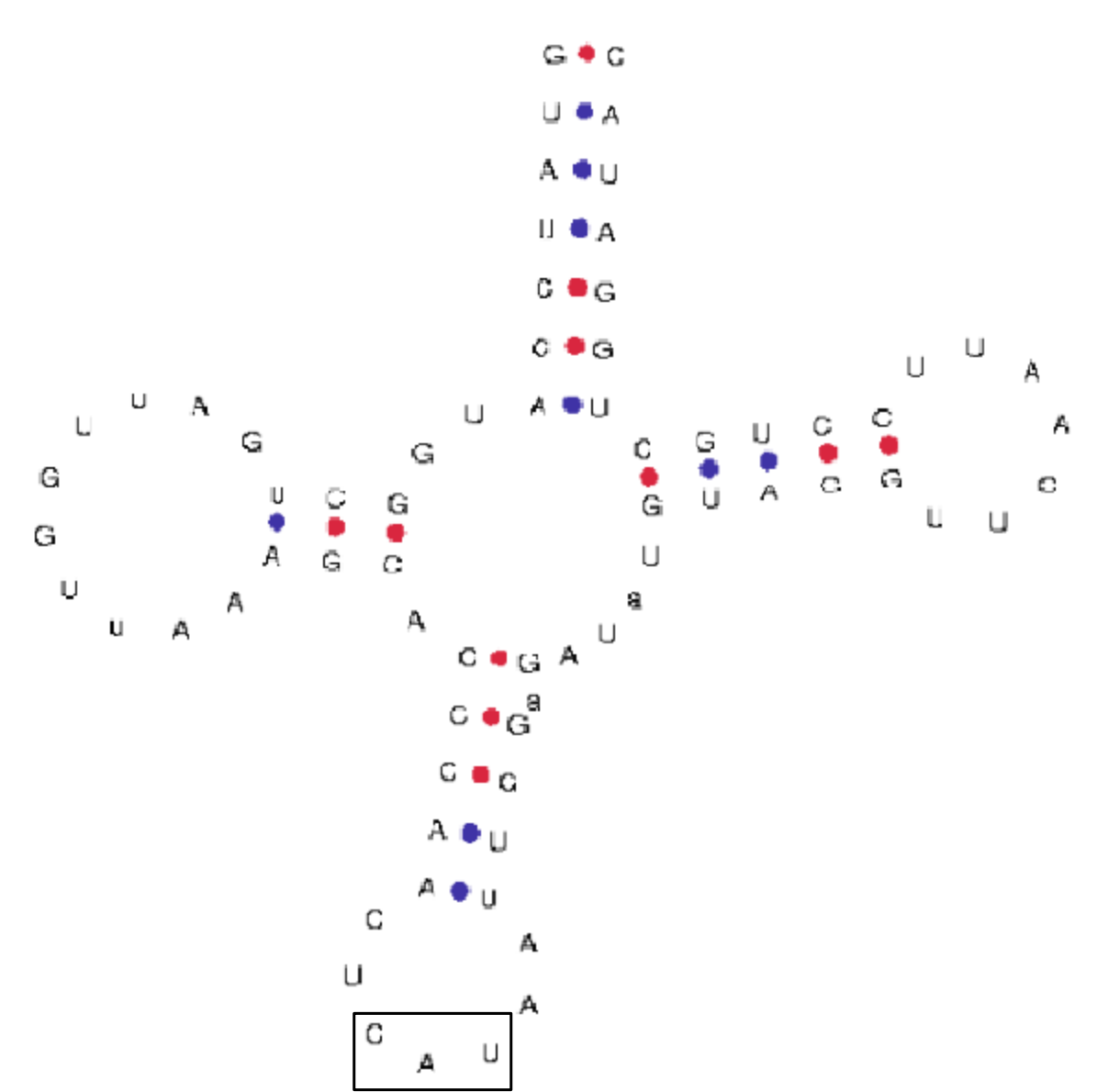

trnW-CCA

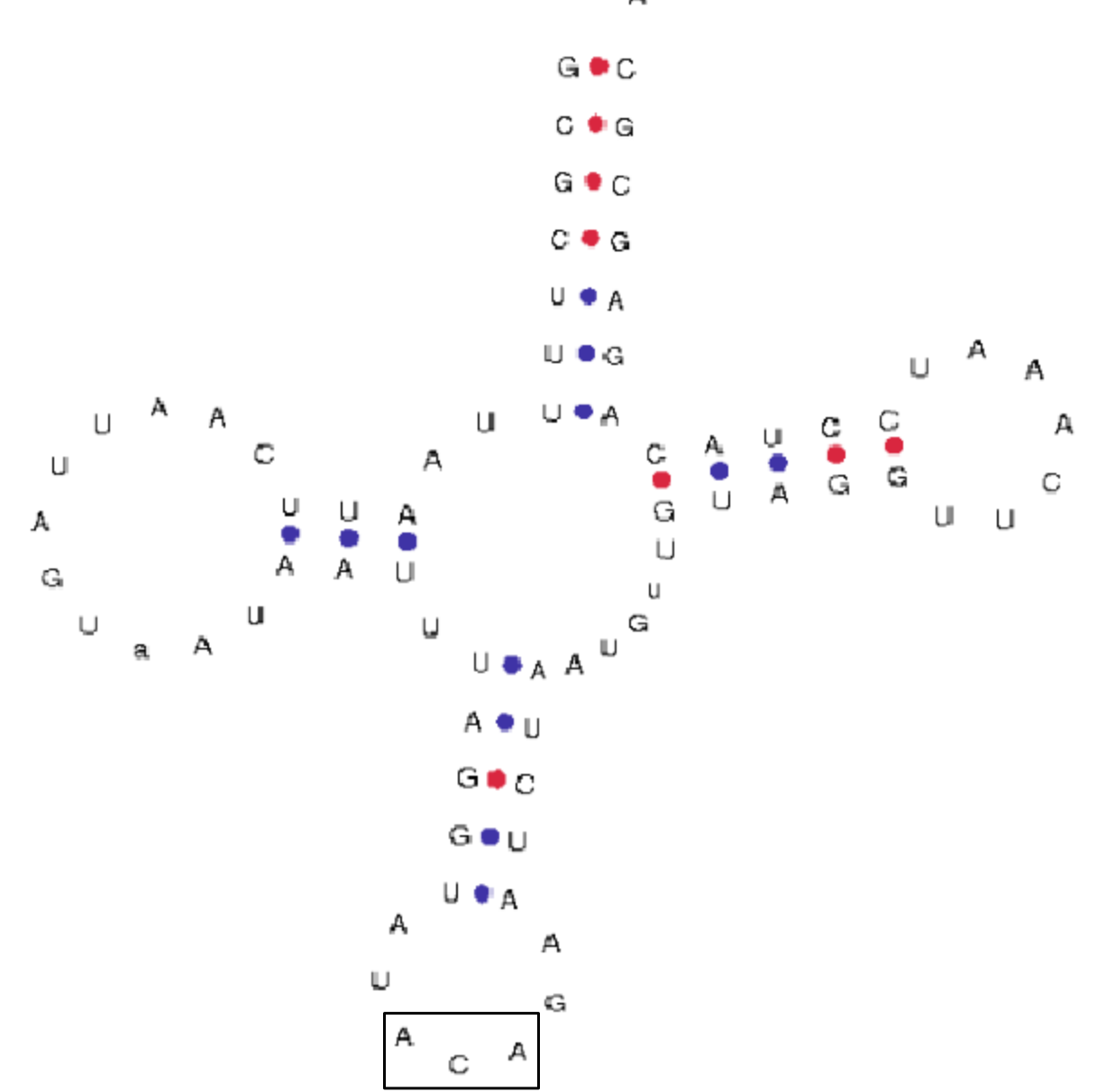

trnY-GUA

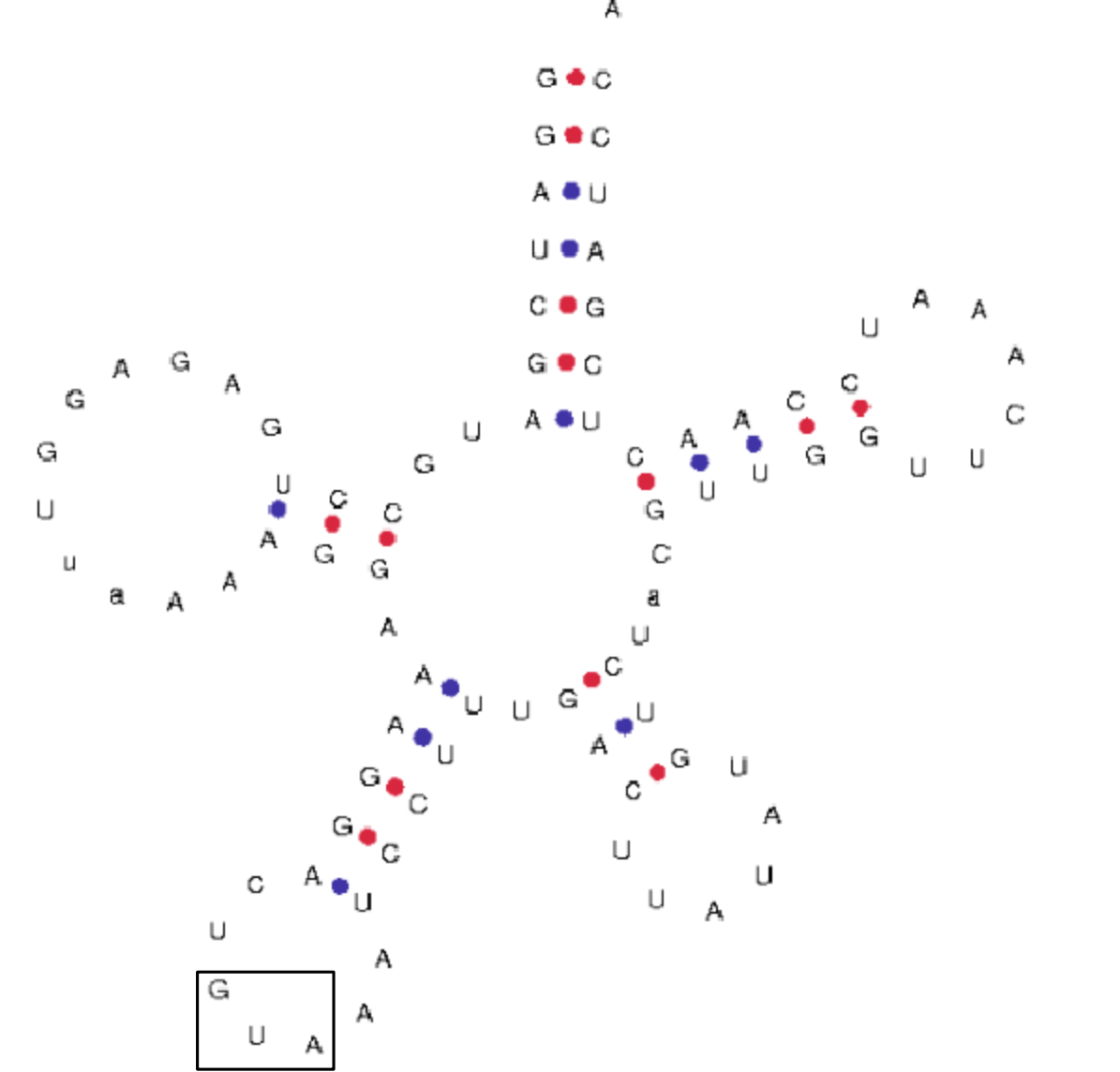

Supplement: Supplementary file 1 [file plants-09-00306-s001.zip › supporting material final proofreading/Figure S1.pdf]

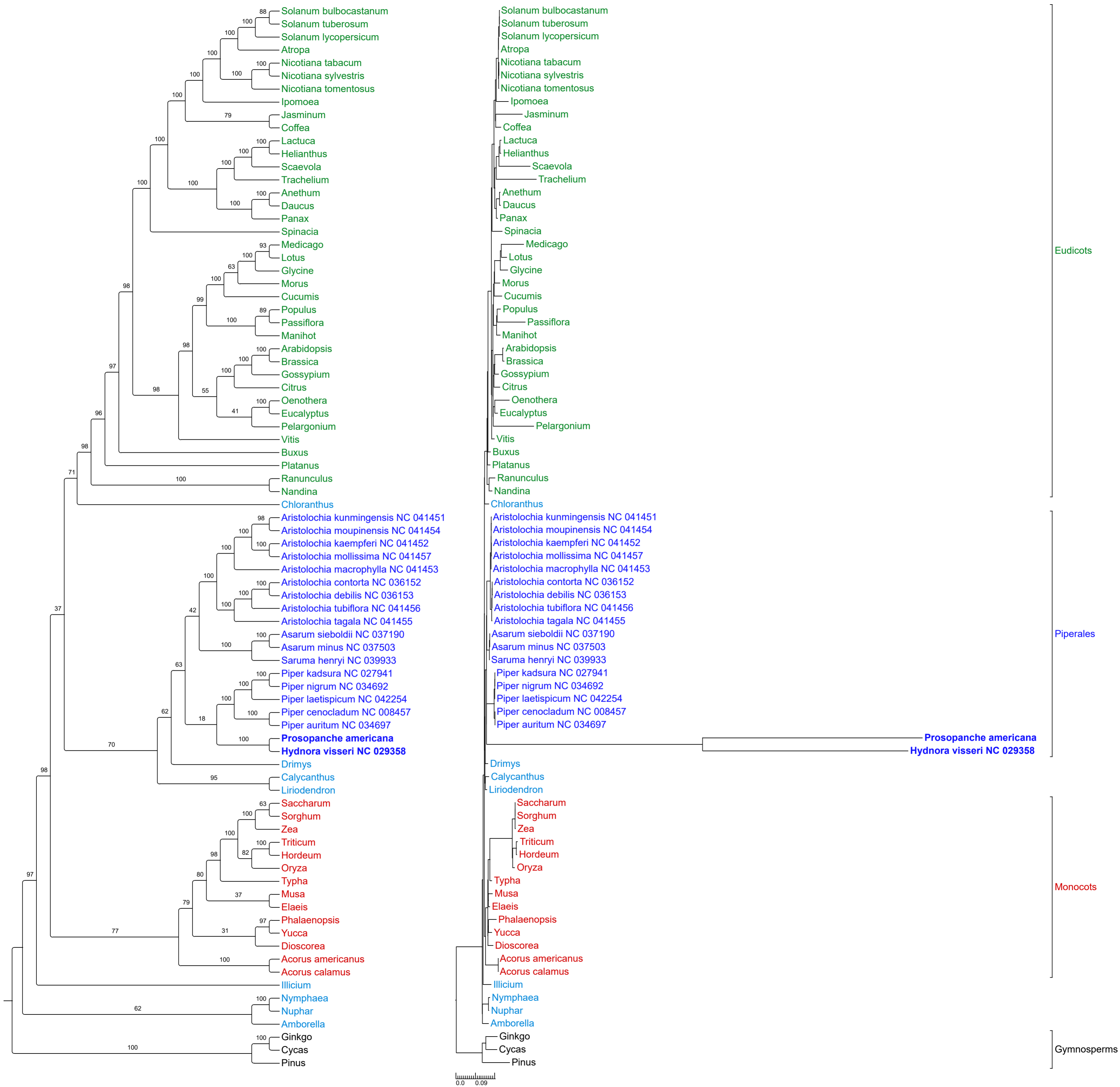

Supplement: Supplementary file 1 [file plants-09-00306-s001.zip › supporting material final proofreading/Figure S2.pdf]

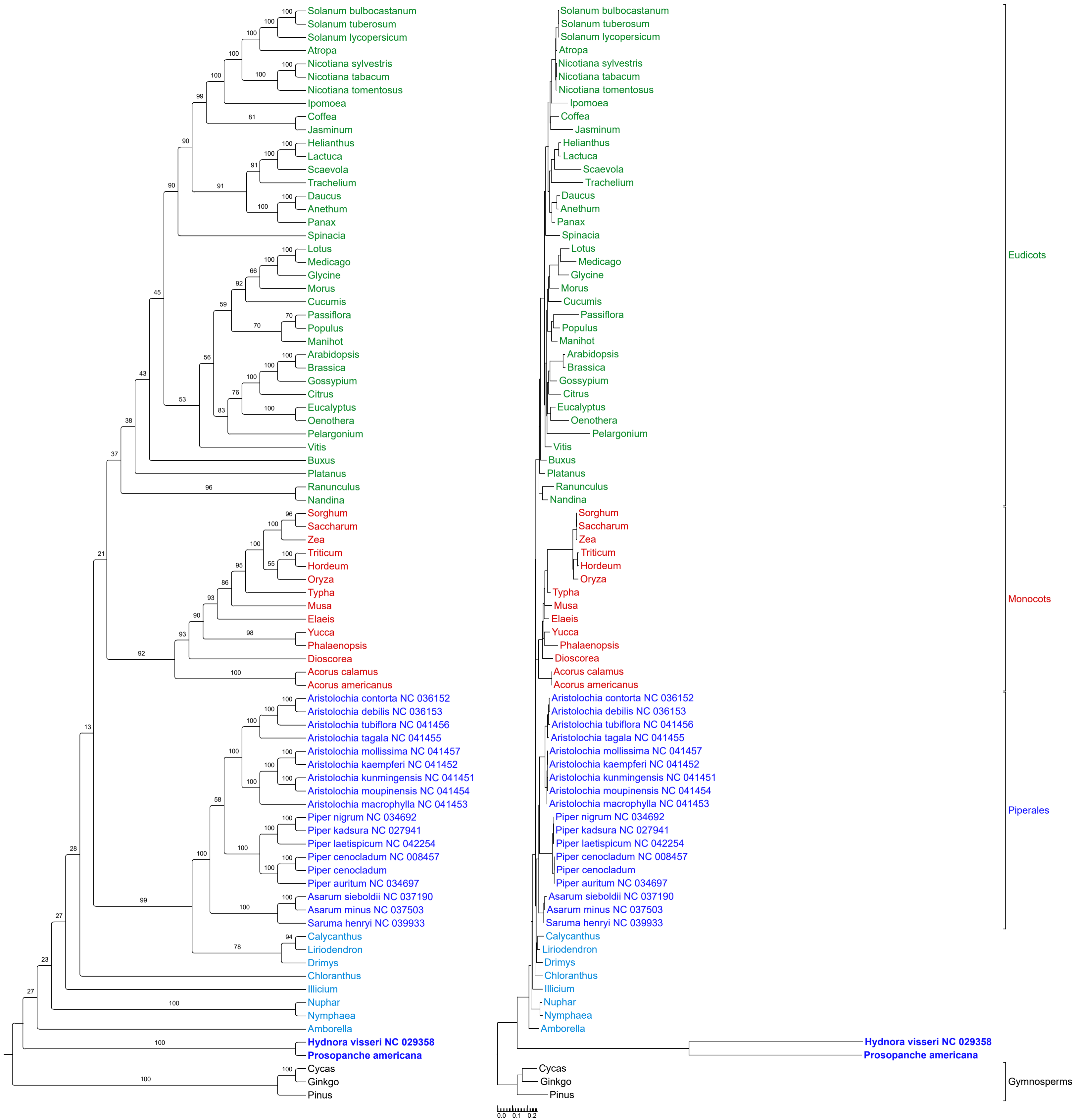

Supplement: Supplementary file 1 [file plants-09-00306-s001.zip › supporting material final proofreading/Figure S3.pdf]

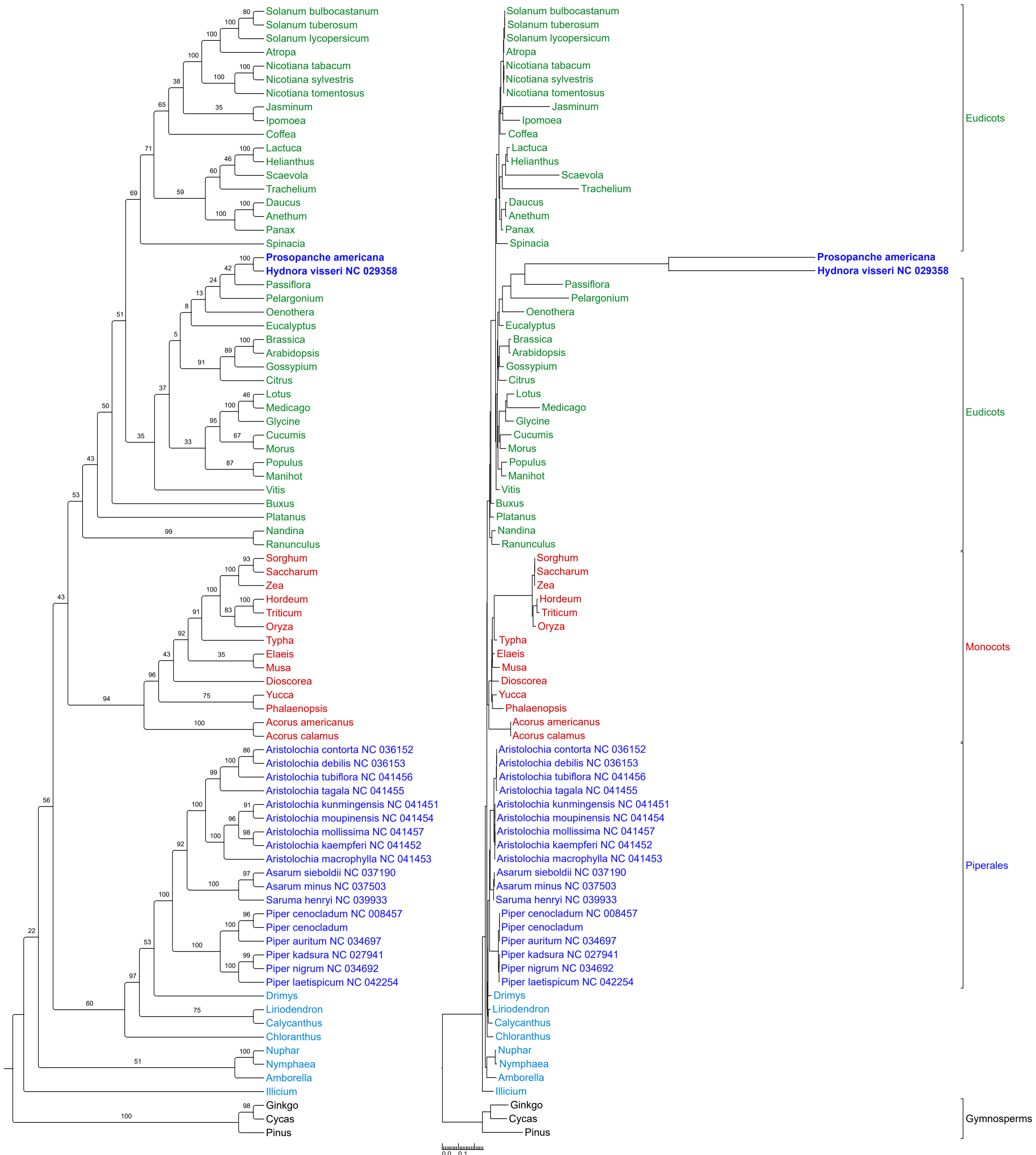

Supplement: Supplementary file 1 [file plants-09-00306-s001.zip › supporting material final proofreading/Figure S4.pdf]
